# A case series study in new restorative surgery in thumb amputation: The Adiposofaciocutaneous flap technique for distal thumb amputation replantation

**DOI:** 10.1016/j.tcr.2024.101052

**Published:** 2024-06-05

**Authors:** Parviz Ahangar, Mohsen Akbaribazm, Mohsen Rahimi, Hosein Pirmohamadi

**Affiliations:** a Shams Tabrizi Clinic, Tehran, Iran; b Department of Basic Medical Sciences, Khoy University of Medical Sciences, Khoy 65371-17636, Iran; c Department of Parasitology and Mycology, School of Medicine, Baqiyatallah University of Medical Sciences, Tehran, Iran; d Trauma Research Center, Baqiyatallah University of Medical Sciences, Tehran, Iran

**Keywords:** Amputation, Thumb finger, Adiposofaciocutaneous flap, Nail

## Abstract

Thumb distal amputation refers to the loss of a portion of the thumb at or near the tip, which can be caused by various injuries such as crush injuries, lacerations, or avulsions. Several surgical methods can be used to repair thumb distal amputations, including composite graft, flap reconstruction, replantation, and amputation revision. In this case report, we describe a successful surgical procedure performed on three healthy men (19, 26, and 44 years old) who suffered a sharp amputation of their left and right hands thumb. In one case initial fixation of the amputated part was performed by a general orthopedic surgeon as a composite graft, two other cases were referred us without any procedure. The procedure involved irrigation and minimal debridement and deepithelializing the amputated part and fixation it with one or two 1.5 mm steinman pins and repairing the nail bed with7/0 absorbable sutures. An adiposofaciocutaneous flap from the index finger was used to cover the pulp of the thumb and the nail bed, while a full-thickness grafts from the same wrist in one case and medial part of ipsilateral arm in others were used to repair the defect on the dorsal side of the index finger. The wound was dressed, and the sutures were removed after two weeks. The base of the flap was detached from the index finger after three weeks, and the kwires were removed after six weeks. The flap and graft were successfully taken, except for a small part of the tip of the thumb. Two years after the operation, in two patients and 3 months in whom was operated recently, all the patient's thumbs had a reasonable shape and length with minimal nail deformity. The use of an index finger based adiposofaciocutaneous flap and full-thickness graft in these cases allowed for successful reconstruction of the thumb and, improving both function and appearance.

## Introduction

The thumb plays a significant role in hand function, as noted by Sir Charles Bell, who said that “on the length, strength, free lateral motion, and perfect mobility of the thumb depends the power of the human hand” [1]. A critical component of thumb function is opposition, for which length, stability, and sensation are paramount. The thumb is responsible for 40 % of all hand function, and as such, traumatic amputation of the thumb is associated with significant functional deficits [2]. Fingertip amputations are the most common injuries to the upper limb. Among upper limb injuries, thumb reconstruction is most frequently required due to trauma, which can result from a variety of mechanisms, including sharp cuts, avulsions, and crush injuries [3,4]. The Ishikawa classification adapted to distal fingertip amputations categorizes amputations in terms of zones of the fingertip based on the nail, comprising four zones distal to the DIPJ and taking into account the angle of the amputation [5].

In cases of initial traumatic thumb amputation, replantation is the first consideration. However, for non-replantable amputated tips, composite grafting is a simple, time- and cost-effective technique in which the amputated tip is directly sutured onto the proximal stump as a composite graft. The tip is initially nourished by diffusion and later through neovascularization [6]. An alternative method for patients for whom microsurgical replantation is not feasible is the pocket principle, first suggested by Brent in 1979 [7]. The pocket principle can be used to enhance the blood supply and support the survival of the replanted digit by increasing the area of vascular contact. Several successful fingertip non-microsurgical implantations using the pocket technique have been documented in the literature [[8], [9], [10]].

To date, many authors have utilized the contralateral axillary region and abdomen as a pocket site for fingertip amputations. However, shoulder, elbow, arm, and finger stiffness caused by secure fixation were noted when the abdominal pocket site was used. Although reduced shoulder, elbow and arm stiffness were noted after this procedure, phalangeal joint stiffness remained. Therefore, in 2010, Puhaindran et al. described the use of a modified palm subdermal pocket site [8], which has been successfully used for fingertip amputations [9]. However, due to the thumb's shorter length and limited range of motion compared with the other digits, the palmar pocket technique is not suitable for reconstructing the thumb. Instead, cross finger subdermal pocketoplasty from the radial side of the third finger has been reported for type I thumb tip amputation with good results [11,12].

In our opinion, the cross finger subdermal pocketoplasty technique is a good way to pocket very distal fingertip amputations, but it has some limitations in coverage of more proximal amputations. To reconstruct an amputated thumb, we have designed an adiposofaciocutaneous cross finger flap from the mediodorsal side of the proximal phalanx of the index finger. This modification of the Sucur and Radivojevic technique (1985) has the advantage of pocketing a larger area of the distal phalanx of the thumb and is suitable for reconstructing more proximal amputations of the thumb. This flap is composed of multiple layers of tissue, including skin, adipose tissue, fascia, and periosteum, which provide a rich blood supply and allow the flap to be transferred to a new location while retaining its viability. The use of this type of flap can help improve the functional and cosmetic outcomes of reconstructive surgery [2]. Like any surgical procedure, the use of an adiposofaciocutaneous flap can be associated with certain risks and potential complications. Some of the possible complications that may arise from the use of this type of flap include: flap failure, infection, bleeding, nerve damage, and cosmetic deformity [12,13]. In the present cases, we utilized the adiposofaciocutaneous flap to quickly improve and maintain the structure and function of the amputated finger and its nail with minor complications. This new method allowed us to successfully reconstruct the finger and its nail, improving both functionality and cosmetic appearance, when microsurgical replantation cannot be done for any reason.

## Case report

**Fig. 1:**
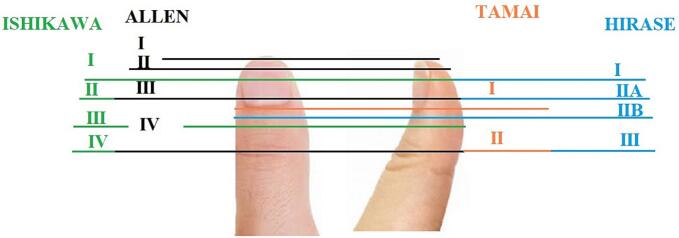
Ishikawa distal fingertip zones: I - beyond mind-nail; II – between mid-nail and nail base (eponychium), III –midway between eponychium and DIPJ, IV – between II and DIPJ.

**Fig. 2:**
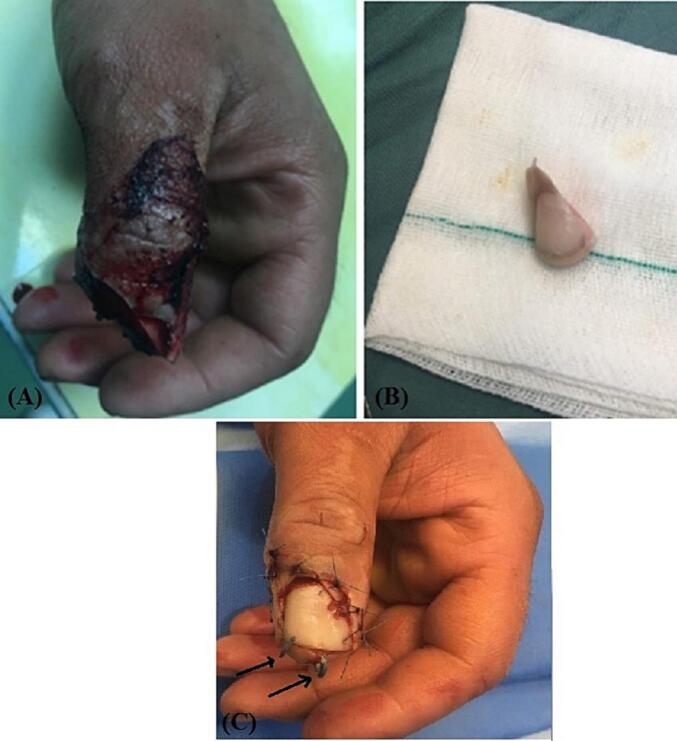
(A) and (B) Oblique type sharp amputation of left-hand thumb like zone II Ishikawa classification. (C) The amputated part of thumb is fixed with two kirshner wires (arrowhead) and simple suture of the skin as composite graft.

Three patients (aged 19, 26, and 44 years) with measurements of 172 ± 12 cm in height, 70.6 ± 5.5 kg in weight, and a body surface area (BSA) of 1.75 ± 0.23 m^2^ were referred to the emergency department in our hospital within first patient 48 h, second 4 h and last patient 10 h after experiencing an avulsion injury resulting in oblique sharp amputation of their right/left-hand thumb. The amputations were classified as zone II according to the Ishikawa classification (Fig. 1, Fig. 2A and B), they had no history of underlying conditions such as diabetes or hypertension, presented with a temperature of 37.4 °C and a blood pressure of 120/80 mmHg. Conscious and alert, the patients reported experiencing severe pain at the site of the lesion. In first case the amputated part of the thumb had been fixed with two Kirschner wires and simple skin sutures as a composite graft by a general orthopedic surgeon 48 h before referring to us (Fig. 2C).

### Operative technique

**Fig. 3:**
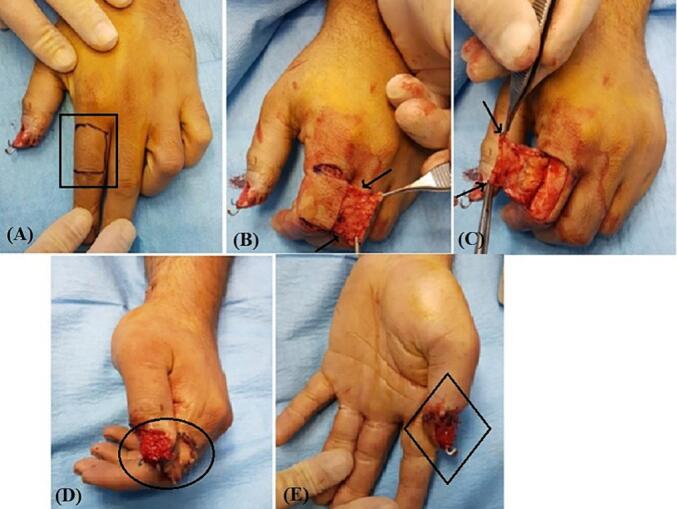
A surgical procedure involving the use of an adiposofaciocutaneous flap was performed to cover the pulp of the thumb and nail bed. (A) The flap area of the proximal part of the index finger was marked with a marker for two transverse incisions (black rectangular box). (B) and (C) A longitudinal incision (red arrowhead) was made on the dorso-ulnar side of the labrum, connecting the transverse incisions. The adiposofaciocutaneous flap was detached (black arrowhead) with an intact base from the side of the index finger and was sutured around the thumb in a rolled fashion. (D) The defect on the dorsal side of the index finger was covered with a full-thickness graft (black oval box) from the volar side of the same wrist. The adiposofascial part was connected to the cutaneous part of the flap from the dorso-radial side. Subsequently, the flap was rotated 180°, and after detaching it from the radial side of the index finger, it was sutured around the thumb in a rolled fashion. (E) The wound was dressed with Vaseline and moist gauze, and the sutures were removed after two weeks. The flap's base was detached from the index finger after three weeks (black rhombus box). (For interpretation of the references to colour in this figure legend, the reader is referred to the web version of this article.)

**Fig. 4:**
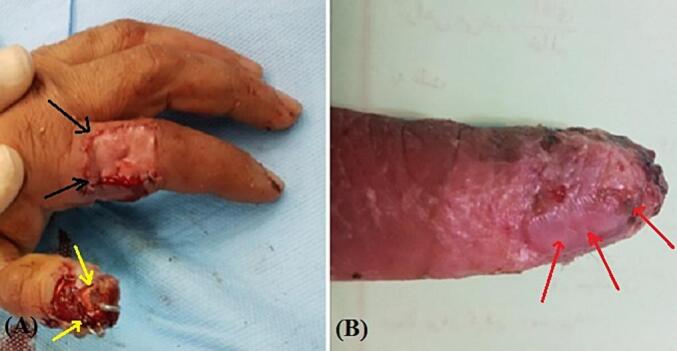
After 4 weeks. (A) and (B) The flap (black arrow) and graft (yellow arrow) were successfully taken except small part of tip of the thumb (red arrow). (For interpretation of the references to colour in this figure legend, the reader is referred to the web version of this article.)

**Fig. 5:**
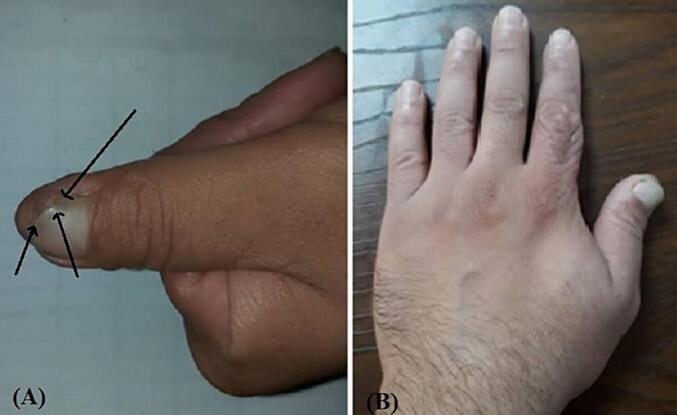
(A) and (B) 2 years after operation the thumb with minimal nail deformity (black arrow).

**Fig. 6:**
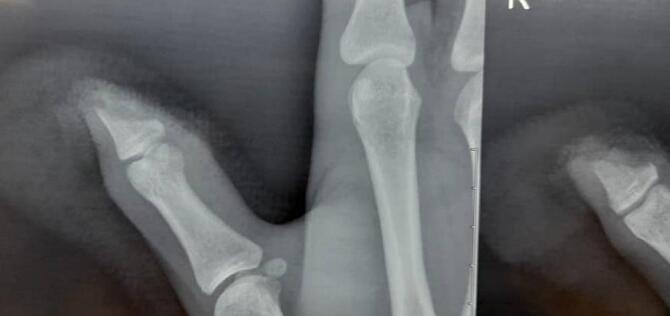
A degree of bone resorption can occur despite graft survival followed by a plain radiography (A-P).

**Fig. 7:**
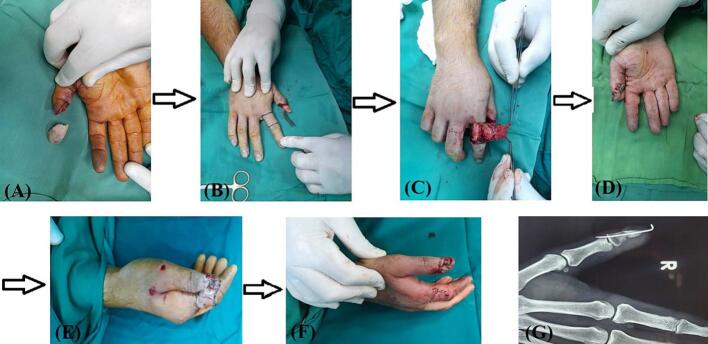
(A) Describing the procedure for replanting a distal thumb following a sharp, oblique amputation using an adiposofaciocutaneous flap technique. (B) Marking the flap area on the proximal part of the index finger for two transverse incisions. (C) and (D) Detaching the adiposofaciocutaneous flap with an intact base from the side of the index finger and suturing it rolled around the thumb. (E) Covering the defect on the dorsal side of the index finger with a full-thickness graft from the volar side of the same wrist, connecting the adiposofascial part to the cutaneous part from the dorso-radial side. (F) Dressing the wound with Vaseline and moist gauze, removing sutures after two weeks, and detaching the flap's base from the index finger after three weeks. (G) A plain radiography (P-A) after 4 weeks.

After irrigation with saline and minimal debridement of thumb and its amputated part, we removed the nail and deepithelialized the amputated part of thumb. In first case we did not remove the pins that were applied by first surgeon. In two cases that referred to us without any procedure the deepithelialized amputated part of thumbs were fixed with one 1.5 mm steinman pin. The nail bed was then repaired using 7/0 absorbable sutures (Fig. 2C). We designed an adiposofaciocutaneous flap from the mediodorsal side of the proximal phalanx of the index finger. The fat tissue on the superficial fascia of the thumb is preserved (for better blood supply) and only the skin flap is separated from the tendons of the extensor muscles. The dermal part of the flap was used to cover the pulp of the thumb, while the adiposofascial part covered the nail bed. Two transverse incisions were made, one aligning with the distal web between the second and third fingers and the other proximal to the skin folds of the proximal interphalangeal (PIP) joint, extending towards the extensor tendon of the index. Additionally, a longitudinal incision was made on the dorso-ulnar side of the proximal phalanx of index finger, connecting the transverse incisions. This incision only involved the dermis area and did not the adiposofascial part. The adiposofascial part of flap encompassed all the subcutaneous tissue on the ulnar side of the proximal of the index finger along with transverse incisions, except for a thin portion of the subcutaneous fat in the ulnar region of the index finger. This segment was removed from the subcutaneous tissue, extending from the ulnar side of the proximal phalanx of the index finger to the neurovascular area of the ulnar side of the index finger. In this case, the adiposofascial part was connected to the cutaneous part of the flap from the dorso-radial side (Fig. 3A-C). Subsequently, the flap was rotated 180°, and it was sutured around the thumb in a rolled fashion, as the cutaneous part covered the pulp of the amputated part and adiposofascial part covered the nail bed. The defect on the dorsal side of the index finger was then grafted using a full-thickness graft obtained from the volar side of the same wrist. Finally, the wound was dressed with Vaseline and covered with moistened gauze (Fig. 3D and E). After two weeks, the sutures were removed, and after three weeks, the base of the flap was detached from the index finger (Fig. 4A). After six weeks, the Kirschner wires were removed. The flap and graft were successfully taken, with the exception of a small part of the tip of the thumb (Fig. 4B). Two years after the operation, the thumb has a reasonable shape with minimal nail deformity (Fig. 5). Two years after operation in two patients and 3 6 months in recently operated case, the thumbs have reasonable shapes with minimal nail deformity (Fig. 5). The patients that were operated earlier had protective sensation but for the last one who was operated 3 months ago we have to wait for assessing of sensation. Minimal degree of bone resorption can occur despite graft survival followed by a plain radiography (Fig. 6, Fig. 7). This method can be a useful alternative to microscopic transplant surgery in centers that do not have the facilities and experience and when the vascular condition is not suitable for repairing. This technique is not suitable for more proximal replants. Such cases should be promptly referred to a tertiary center with microsurgical capabilities, especially if microsurgery is not available locally.

## Discussion

Several methods have been used to maintain the appearance of a normal finger after fingertip amputations. Replantation gives the best results but may not be possible in some patients and circumstances [14]. When replantation is not possible, reconstructive options include composite grafts, pedicle adipofascial turnover or skin flaps, skin grafts, simple closure or secondary intention, and, depending on the circumstances, toe transfer [15]. Composite grafting has been widely performed for distal fingertip amputations, but variable success rates are reported throughout the literature, with key complications being infection and necrosis [16]. While composite grafting has a high success rate and produces good results in treating non-replantable fingertip amputations in pediatric patients, its success rate in adults can be as low as approximately 20 % [17]. The Hirase technique cools the grafted stump at 4 °C in the first 72 h, without shortening it. Ice and aluminum foil are used to keep the graft cool until neovascularization occurs, ensuring the survival of the composite graft [18,19].

To increase the survival of the grafted stump and preserve the shape and function of the fingernail, several techniques have been published based on the removal of fat from the stump, bone resection, and connections with local flaps, which decrease the graft volume for plasmatic diffusion. However, these techniques often result in shortening of the finger and nail atrophy and deformities [20,21]. Another technique involves the removal of all soft tissues, except for the nail and bone of the amputated part of the finger, and coverage with a homo-digital neurovascular anterograde island flap as a reposition flap for fingertip reconstruction [22]. A dorso-ulnar flap combined with periosteum harvested from the first metacarpal was used to cover the volar bone of the thumb, resulting in good functional and cosmetic outcomes [23].

Palmar pocketing for fingertip amputations has been used to avoid the problems associated with other techniques, with good success rates reported. Arata et al. (2001) replaced the thoracic subcutaneous pocket with an ipsilateral palmaris, which is more convenient for patients, especially children. The study reported full reintegration in 81 % of the treated patients, with 19 % of patients exhibiting a small area of necrosis around the tip. Two patients required removal of the exposed bone, and in one patient, there was spontaneous healing of the exposed bone, but with no total loss [11]. While palmar pocketing is a good way to pocket very distal fingertip amputations, it has limitations in coverage of more proximal amputations. In our opinion, replantation of thumb tip amputations with adiposofaciocutaneous flap pocketing from the dorso-ulnar side of the index finger can be a good alternative procedure to save the amputated part of the thumb. In this method, unlike the pactoplasty method in the abdomen, the hand is free and does not restrict movement in the wrist and elbow and shoulder jonts and noninvolved fingers are free for daily use. While this method offers numerous advantages, the probability of restoring sensation in the area may be lower compared to microscopic surgery due to its novelty.

## Conclusion

In cases of amputation, an adiposofaciocutaneous flap may be used to cover the pulp of the thumb and the nail bed. This type of flap is composed of multiple layers of tissue, including skin, adipose tissue, fascia, and periosteum, which provide a rich blood supply and allow the flap to be transferred to a new location while retaining its viability. The use of this type of flap can help improve the functional and cosmetic outcomes of reconstructive surgery following amputation.

## CRediT authorship contribution statement

**Parviz Ahangar:** Methodology, Investigation, Formal analysis, Data curation, Conceptualization. **Mohsen Rahimi:** Writing – review & editing, Writing – original draft, Validation, Software. **Hosein Pirmohamadi:** Writing – original draft, Visualization, Software, Methodology, Investigation, Formal analysis, Data curation, Conceptualization.

## Declaration of competing interest

The authors declare that there is no conflict of interest.

## Data Availability

All data associated with the article is available if required.
